# Induction of allergen-specific blocking IgG using patch delivered recombinant Bet v 1 in guinea pigs

**DOI:** 10.1186/2045-7022-4-S2-O18

**Published:** 2014-03-17

**Authors:** Clarissa Cabauatan, Raffaela Campana, Katarzyna Niespodziana, Christoph Reinisch, Urban Lundberg, Andreas Meinke, Rainer Henning, Angela Neubauer, Rudolf Valenta

**Affiliations:** 1 Medical University of Vienna, Dept. of Pathophysiology and Allergy Research, Vienna, Austria; 2 Intercell Austria AG Vienna, Austria; 3 Biomay AG, Vienna, Austria

## Background

Allergen-specific immunotherapy (SIT) is the only specific, disease-modifying treatment for allergy and may have long-lasting effects. It is given traditionally in the form of multiple subcutaneous injections which makes the treatment inconvenient. Furthermore, systemic allergen-administration may cause severe side effects. Therefor the development of alternative routes for SIT has been a long-sought goal. The aim of this study was to investigate if epicutaneous application of recombinant birch pollen allergen Bet v 1 (i.e., patch vaccination) can induce systemic allergen-specific IgG responses with protective activity.

## Methods

Groups of outbred female Dunkin Hartley guinea pigs (GP) (10 animals per group) were immunized subcutaneously with aluminium hydroxide-adsorbed rBet v 1 (5 µg), aluminium hydroxide alone (negative control) or by patch delivery system (PDS) using two doses of rBet v 1 (30 µg or 100 µg per patch) on days 1, 15, 28 and 43. ELISA experiments were performed to measure the development of IgG specific for Bet v 1, Bet v 1-related pollen and food allergens ( i.e., Aln g 1, Cor a 1, Mal d 1) and for unfolded Bet v 1 fragments. Furthermore, guinea pig antibodies were tested in IgE ELISA inhibition experiments with allergic patients' sera to study whether allergen-specific IgG can block allergic patients allergen-specific IgE binding.

## Results

Already after 42 days and three s. c. injections of aluminium hydroxide-adsorbed rBet v 1 or three patch test vaccinations with the high dose PDS in combination with LT, a relevant induction of allergen-specific IgG was observed in more than 20% of the outbred animals. More importantly, these antisera inhibited allergic patients' (n=5) IgE binding to Bet v 1. Interestingly, we noted that only vaccination with aluminium hydroxide-adsorbed rBet v 1 induced IgG antibodies against sequential epitopes and cross-reactive IgG antibodies whereas patch vaccination seemed to induce primarily Bet v 1-specific IgG directed against conformational epitopes.

## Conclusion

Our results suggest that patch vaccination with recombinant Bet v 1 may be a promising strategy for SIT against birch pollen allergy. This study was supported by grant F4605 of the Austrian Science Fund and a research grant from Biomay AG, Vienna, Austria.

